# TAR-VIR: a pipeline for TARgeted VIRal strain reconstruction from metagenomic data

**DOI:** 10.1186/s12859-019-2878-2

**Published:** 2019-06-04

**Authors:** Jiao Chen, Jiating Huang, Yanni Sun

**Affiliations:** 1 Computer Science and Engineering, Michigan State University, East Lansing, 48824 USA; 2 Institute of Clinical Pharmacology, Guangzhou University of Chinese Medicine, Guangzhou, 510006 China; 3 Electronic Engineering, City University of Hong Kong, Hong Kong, China

**Keywords:** RNA virus, Read classification, Strain assembly, Viral metagenomics

## Abstract

**Background:**

Strain-level RNA virus characterization is essential for developing prevention and treatment strategies. Viral metagenomic data, which can contain sequences of both known and novel viruses, provide new opportunities for characterizing RNA viruses. Although there are a number of pipelines for analyzing viruses in metagenomic data, they have different limitations. First, viruses that lack closely related reference genomes cannot be detected with high sensitivity. Second, strain-level analysis is usually missing.

**Results:**

In this study, we developed a hybrid pipeline named TAR-VIR that reconstructs viral strains without relying on complete or high-quality reference genomes. It is optimized for identifying RNA viruses from metagenomic data by combining an effective read classification method and our in-house strain-level de novo assembly tool. TAR-VIR was tested on both simulated and real viral metagenomic data sets. The results demonstrated that TAR-VIR competes favorably with other tested tools.

**Conclusion:**

TAR-VIR can be used standalone for viral strain reconstruction from metagenomic data. Or, its read recruiting stage can be used with other de novo assembly tools for superior viral functional and taxonomic analyses. The source code and the documentation of TAR-VIR are available at https://github.com/chjiao/TAR-VIR.

**Electronic supplementary material:**

The online version of this article (10.1186/s12859-019-2878-2) contains supplementary material, which is available to authorized users.

## Background

Pathogenic human viruses such as human immunodeficiency virus (HIV), hepatitis C virus (HCV), Severe Acute Respiratory Syndrome (SARS) coronavirus (SARS-CoV), and H1N1 flu virus, still claim millions of lives each year despite centuries studies of the vaccine and treatment [1, 2]. Thus, characterizing human viral pathogens, including recognizing novel ones, remains crucial. Development of the next-generation sequencing (NGS) technologies sheds lights on characterizing the virus composition in both natural environmental and clinical samples. In particular, viral metagenomic sequencing, which allows us to circumvent the need for virus isolation and cultivation, can conduct comprehensive sequencing of all viruses in a sample. Thus, multiple viruses, including new ones, can be identified in viral metagenomic sequencing data.

Today, viral metagenomic data have become the primary source of virome analysis and virus discovery [3]. For example, in order to test whether increased levels of anelloviruses or other viruses in plasma are associated with higher levels of persistent T-cell activation during anti-retroviral therapy (ART), Li et al. detected all viruses using metagenomic data of plasma samples from 19 adults on effective ART [4]. Lim et al. characterized the gut virome and bacterial microbiome in a longitudinal cohort of healthy infant twins in order to study the dynamics of eukaryotic RNA and DNA viruses during the first years of life [5]. There are also global-scale studies on viruses in natural environmental samples such as ocean water [6, 7]. In addition to these examples, a more comprehensive review about the studies using viral metagenomic data in diagnostics, surveillance and outbreak source tracing, and biodiversity studies can be found in [8].

We are particularly interested in characterizing RNA viruses in metagenomic data because many of them are clinically important. blackRNA viruses can form quasispecies, which contain related but distinct haplotypes generated during RNA virus replication. Different haplotypes can lead to different biological properties such as virulence. Accurate RNA virus characterization needs to assembly the reads into strain-specific contigs for downstream taxonomic and functional analyses. Generic assembly tools are often error prone for metagenomic data and are also resource demanding. Thus, a commonly adopted strategy is to classify reads into different biological entities such as species before conducting assembly.

In this study, our goal is to develop a new pipeline that can classify RNA viral reads and also produce the assembled viral strains (i.e. haplotypes) from classified reads. Various methods and tools have been developed [9–12] to characterize viruses in viral metagenomic data. Despite their promising results in virus identification and discovery, there is room to improve in two aspects. First, read classification in many of these tools rely on read mapping against characterized reference genomes [8], which are not always available for fast mutating RNA viruses. For example, emerging pathogenic viruses such as SARS-CoV could only have local conservation with available genomes. Second, most of these tools adopt generic assembly tools, which cannot distinguish different viral strains.

### Related work

Depending on the required inputs, existing programs for analyzing virus composition can be roughly divided into two groups. One group take assembled contigs as input for functional and compositional analyses. Another group of tools classify reads first and then conduct assembly for classified reads. Below we describe both types of tools and their limitations.

Viral metagenomic analysis tools in group one require metagenomic assembly. For example, Espino et al. [13] identify viral sequences from assembled metagenomic contigs of sizes greater than 5kb. VirSorter [14] detects viruses in assembled contigs at least 3kb. The viral sequences are usually screened by comparing the contigs with a curated set of viral protein families. However, conducting de novo assembly for metagenomic data is still one of the most difficult computational problems. Unknown number of species in a sample and heterogeneous sequencing coverage can lead to erroneous assemblies.

Therefore, most of the viral metagenomic data analysis methods combine reference-based classification and de novo assembly. This strategy classifies reads into different taxonomies or functional groups using reference-based methods and then conducts de novo assembly for reads within the same group. For example, VIP [15], drVM [16], and VirusTAP [17] all apply this strategy. They classify viral reads by either aligning reads to available viral references or removing reads of the host and other species. Next, existing assembly tools such as SPAdes [18] are employed to the virus-like reads to produce the final assembly results. While these tools made significant contributions in purifying the data by removing non-virus reads and then classifying virus-like reads into functional/taxonomic groups, their performance heavily depends on the quality of the references.

The limitation of reference genomes poses a critical challenge of applying reference-based tools for RNA virus analysis in metagenomic data. While being regarded as the most abundant biological entities on earth, only a small portion of viruses have been sequenced and characterized. Besides, for RNA viruses with high mutation rates, high-quality reference genomes of a viral population are not always available. For example, many emerging viral diseases are caused by zoonotic viruses, which originate in vertebrates but can infect human. The genomes of some emerging viruses may only share medium sequence identity with their peers in animals, creating difficult circumstances for reference-based read classification.

For RNA virus characterization, related tools also include haplotype reconstruction pipelines designed to assemble viral strains in quasispecies. A majority of these tools are reference-based and take the alignments of reads against reference genomes as input. HaploClique [19], ViQuas [20], VGA [21] all belong to this group.

### Overview of our work

Here we introduce TAR-VIR, which provides a useful addition to existing tools for identifying targeted RNA viruses and their haplotypes in metagenomic data. The “targeted” viruses are those that still possess local sequence similarity with the reference genomes. A completely new virus that does not share any conservation with any reference genome won’t be detected by our method.

Our pipeline combines reference-based strategy and de novo assembly and is optimized for the following applications. 1) Identifying host-switching viruses such as SARS-CoV using remotely related viruses in other hosts as the references. 2) Reconstructing viral haplotypes that are divergent from a known virus family. 3) Recovering viruses and their genomes that contain genes or functional sites of interest to users. TAR-VIR is faster and more effective in identifying targeted viruses than applying generic assembly programs to the whole metagenomic data set. This is particularly important for large and complicated metagenomic data sets containing a small percentage of viruses. Meanwhile, TAR-VIR is more tolerant to incomplete or low-similarity references than existing reference-based tools.

We applied TAR-VIR to a simulated metagenomic data set containing five haplotypes of SARS-CoV and a real human blood plasma metagenomic data set. The comparisons with both de novo assembly tools and reference-based haplotype reconstruction tools demonstrated the utility of TAR-VIR in recovering RNA viruses from metagenomic data with limited references.

## Results

We have developed a modular structured tool named TAR-VIR for reconstructing viral haplotypes from metagenomic data. The final outputs of this tool are assembled viral contigs corresponding to different strains. We focus on evaluating the performance of the read recruiting stage and also its impact on the final assembly.

### Exp1: reconstruct the SARS haplotypes using the bat coronavirus as the reference

In this experiment, we mimic the scenario in which SARS-CoV [22] is an emerging virus infecting humans. Our goal is to reconstruct the SARS-CoV haplotypes using other coronaviruses as references. During the breakout of SARS, electron microscope image reveals the crown-like shape of the infectious agent, providing clues to use coronaviruses as references.

To test this, we assume that the bat coronavirus (NC_014470.1) was sequenced and available to use as a reference, although it was actually sequenced after the breakout of SARS.

#### Data properties and evaluation metrics

A viral metagenomic dataset containing Influenza (NC_002023.1), hepatitis C virus (HCV, NC_004102.1), and 5 SARS-CoV haplotypes, was simulated. The SARS-CoV haplotypes were created from the SARS-CoV reference (NC_004718.3) genome by mutating bases at randomly selected locations. The sequence similarity between any two haplotypes is above 96%. The abundance of each haplotype is calculated based on a power law equation [23]. The total sequencing depths for the 5 SARS-CoV haplotypes are 1000-x, with 438-x, 219-x, 146-x, 109-x, and 88-x for each haplotype, respectively. The sequencing depths for Influenza and HBV are 700-x and 300-x, respectively. All the data sets were simulated by ART-illumina [24] as error-containing MiSeq paired-end reads, with the read length of 250 bp, the average insert size of 600 bp, and the standard deviation of 150 bp. In total, there are 173,703 simulated reads, of which 119,002 reads are from the five SARS-CoV haplotypes.

With the available bat coronavirus as the reference, the simulated reads were aligned with both Bowtie2 [25] and BWA [26]. We then applied the overlap extension component of TAR-VIR to isolate and enrich SARS-CoV reads, and assembled them with de novo assembly tools. Both the read recruitment and the assembly performance were evaluated. For the simulated data, the ground truth of the originating haplotype and position of each read is known. Thus read recruiting performance can be evaluated using the reads’ positions and originating haplotypes. In summary, we examine how many reads are correctly recruited for each haplotype and report the haplotype coverage and depth.

The assembly performance was evaluated using the known genomes of the 5 SARS-CoV haplotypes and MetaQuast [27]. Similar to other works, we quantified the assembly continuity, completeness, and accuracy in terms of number of contigs, N50, genome coverage, and mismatch rate. N50 is defined as the maximal length so that all contigs above this length contain at least 50% of all the contig bases. Genome coverage is the percentage of the five haplotypes’ genomes being aligned by at least one contig. Mismatch rate is the percentage of mismatches between the aligned contigs and the references. In all cases, contigs of at least 500 bp are aligned to the viral reference sequences for evaluation. The assembly results were also benchmarked with other popular assembly tools SGA [28], SPAdes [18], and SAVAGE [29].

#### Performance of read recruitment

We applied both Bowtie and BWA in the read mapping stage. By adjusting the scoring function related parameters, we constructed different sets of seed reads that can be aligned to the references with different approximate match constraints. For each seed set, the recruited reads generated by TAR-VIR with overlap cutoff 150 are recorded. Table 1 compares the aligned and recruited reads for each SARS-CoV haplotype. Besides approximate match rates, we also considered local and “glocal” alignment mode, where the glocal mode requires the end-to-end alignment of the read against the reference. Using local alignment mode for read mapping can usually produce a larger seed set. However, it is possible that some of the locally aligned reads are not sequenced from the underlying haplotypes. In Table 1, we used local alignment mode for BWA and glocal model for Bowtie. Thus, the seed sets constructed by BWA is larger than Bowtie.

**Table 1:** Read recruitment results by using seed sets constructed with Bowtie2 and BWA

Bowtie2	Alignment										
	Number	Depth				Coverage					
		h1	h2	h3	h4	h5	h1	h2	h3	h4	h5
L,0,-0.3	55	0.13	0.01	0.06	0.15	0.12	0.01	0.01	0.01	0.01	0.01
L,0,-0.6	925	3.6	1.5	0.9	0.9	0.9	0.07	0.06	0.05	0.07	0.08
L,0,-0.9	8154	32	14	9	8	7	0.31	0.31	0.27	0.27	0.3
L,0,-1.2	13,221	49	24	15	13	10	0.43	0.45	0.42	0.44	0.43
	Recruitment										
	Number	Depth				Coverage					
		h1	h2	h3	h4	h5	h1	h2	h3	h4	h5
L,0,-0.3	45,504	182	89	59	41	11	1.0	1.0	1.0	1.0	0.37
L,0,-0.6	46,576	183	90	60	42	18	1.0	1.0	1.0	0.96	0.55
L,0,-0.9	52,337	198	96	63	46	37	1.0	1.0	1.0	0.98	0.99
L,0,-1.2	55,485	182	89	59	41	39	1.0	1.0	1.0	1.0	0.99
BWA	Alignment										
	Number	Depth				Coverage					
		h1	h2	h3	h4	h5	h1	h2	h3	h4	h5
B:8	24,585	89	46	28	20	18	0.4	0.37	0.33	0.31	0.34
B:4	41,564	152	78	50	37	32	0.63	0.57	0.56	0.57	0.53
B:2	51,995	195	94	63	46	39	0.79	0.78	0.77	0.70	0.74
B:1	52,688	199	94	64	47	39	0.81	0.78	0.80	0.71	0.76
	Recruitment										
	Number	Depth				Coverage					
		h1	h2	h3	h4	h5	h1	h2	h3	h4	h5
B:8	62,609	235	117	75	55	44	1.0	1.0	1.0	1.0	0.99
B:4	72,901	270	135	89	65	53	1.0	1.0	1.0	1.0	1.0
B:2	79,755	299	146	97	71	58	1.0	1.0	1.0	1.0	1.0
B:1	78,540	294	143	96	70	57	1.0	1.0	1.0	1.0	1.0

The “Alignment” section contains results for aligned reads. The “Recruitment” section contains results for recruited reads by TAR-VIR using the aligned reads. For each row, the aligned reads in “Alignment" section are the seed set for the recruited reads in “Recruitment” section. For Bowtie2, the “–score-min” parameter was set to allow different alignment error rates corresponding to 5%, 10%, 15%, and 20%, respectively. For BWA, “-A” is fixed as its default value 1. “-B” was modified to allow different error rate similar to Bowtie2. “Number” is the number of aligned or recruited reads. “Depth” is the average sequencing coverage. “Coverage” is the percentage of genome covering by at least one read. h1 to h5 represent the five SARS-CoV haplotypes

**Fig. 1:**
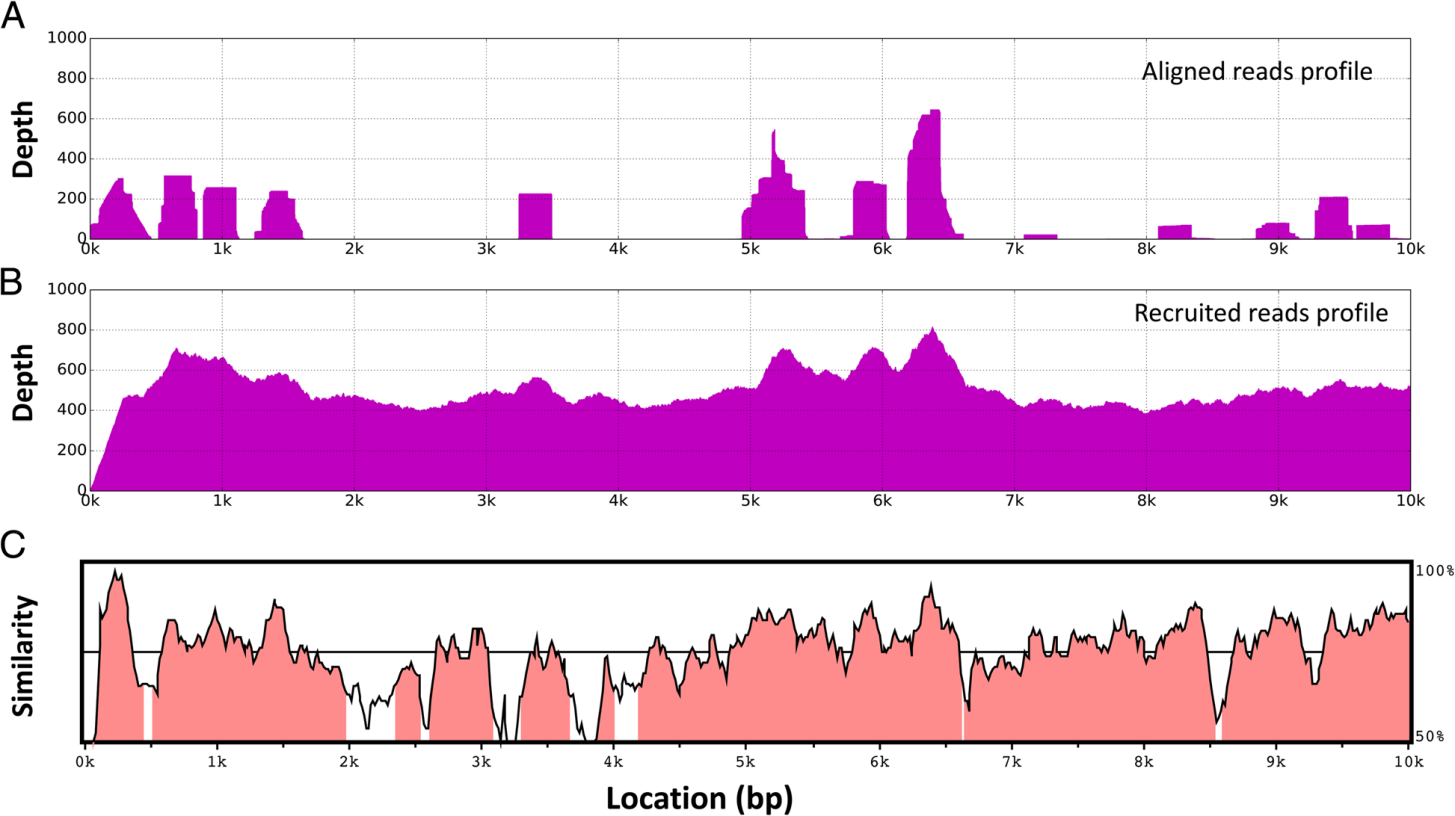
Enriching SARS-CoV reads using the bat coronavirus genome as the reference. **a** and **b** show the aligned and recruited reads profile. The dataset was aligned by BWA with the default parameter ("-B 4, -A 1"). BWA is chosen to include more locally aligned reads in (**a**). The reads were recruited using the overlap cutoff of 150 bp. **c** displays the sequence identity between SARS-Cov and the bat coronavirus. The profile was generated using VISTA [41]

Even with the least stringent threshold, the aligned reads have lower genome coverage than recruited reads, which is expected because SARS-CoV does not share genome-scale high similarity with the bat coronavirus (Fig. 1c). In particular, with the parameter “-B 1", BWA can align slightly more reads than what Bowtie2 can recruit with the parameter “L, 0, -0.9" ((52,688 vs. 52,377). The recruited reads (52,377), however, cover 20%-30% more genomes for the five haplotypes. This indicates that alignment-based methods tend to identify reads sequenced from highly similar regions between the target and the reference viruses, while the recruitment method is more likely to obtain reads from the whole genome of the target viruses. Worth noting is all the recruited reads are from SARS-CoV (no contamination from Influenza and HBV).

Figure 1a and b compared the genome coverage of seed reads and recruited reads. Directly aligning the reads to the bat coronavirus covers only a small proportion of the whole genome (Fig. 1a), leading to incomplete assembly. Using these aligned reads as seeds, TAR-VIR is able to recruit many more reads that nearly cover the whole genome of SARS-Cov, as shown in Fig. 1b.

Table 1 also shows that the numbers of recruited reads do not heavily rely on the number of the seed reads. Even when the seed set is small (e.g. the seed sets constructed using Bowtie2), many new reads can be recruited during each iteration. After multiple iterations, the final set of recruited reads can be significantly larger than the seed set, bounded by the sequencing depth of the haplotypes. On the other hand, if the seed sets contain many reads from non-relevant species, the final set of recruited reads could even include all the reads from the input, which makes the read recruiting useless. Because of this, we prefer to construct the seed set using glocal mode to ensure high quality.

#### Recruited reads lead to better assembly performance

Both the aligned and recruited reads were assembled with de novo assembly tools. PEHaplo is the default assembly component in TAR-VIR. As TAR-VIR has a modular structure, other de novo assembly tools including SGA, SPAdes, and SAVAGE are also used to replace PEHaplo for haplotye reconstruction. SPAdes was run with –meta option, which is same as metaSPAdes [30]. As the aligned reads cover at most 80% of the genomes even with the least stringent alignment threshold, it is not proper to apply the conventional reference-based assembly methods for this data set.

The complete de novo assembly results using both aligned and recruited reads are presented in Additional file 1: Table S1. Part of the results are shown in Table 2 due to space limiation. For all assembly tools, using recruited reads produces better results: longer contigs and higher genome coverage. Significantly, this is not simply due to the increased number of reads. For example, as shown in Table 2, the reads recruited by Bowtie2 with parameter “L, 0, -0.9” is less than BWA-aligned reads when B is 1 (52,337 vs. 52,668). But the recruited reads produce contigs at least ten times longer than the aligned reads, and twice higher in the genome coverage. By comparing the assembly performance of all tested tools on the recruited reads, our assembly component PEHaplo consistently has higher N50 and genome coverage than others. Overall, PEHaplo and SGA perform better than the other two assembly tools.

**Table 2:** Assembly results on SARS-CoV aligned and recruited metagenomic data

Aligned	Tool	# Contigs	N50	Genomes covered (%)	Mismatch rate (%)
Bowtie2 L,0,-0.9	TAR-VIR	58	505	19.7	0.02
	SGA	56	505	20.1	0.03
	SPAdes	34	569	12.9	0.16
	SAVAGE	54	455	17.5	0.0
Recruited	Tool	# Contigs	N50	Genomes covered (%)	Mismatch rate (%)
	TAR-VIR	7	29,676	98.9	0.0
	SGA	13	26,729	98.9	0.0
	SPAdes	14	15,882	92.1	0.51
Bowtie2 L,0,-0.9	SAVAGE	22	12,445	97.0	0.0
Aligned	Tool	# Contigs	N50	Genomes covered (%)	Mismatch rate (%)
	TAR-VIR	84	1192	55.1	0.0
	SGA	85	1027	56.5	0.0
	SPAdes	67	1012	44.6	0.12
BWA B:1	SAVAGE	68	669	32.3	0.0
Recruited	Tool	# Contigs	N50	Genomes covered (%)	Mismatch rate (%)
BWA B:1	TAR-VIR	6	29,706	99.5	0.0
	SGA	18	12,638	99.5	0.0
	SPAdes	21	10,353	89.2	0.39
	SAVAGE	56	5140	89.3	0.0

The default assembly tool in TAR-VIR is PEHaplo. Definition of the metrics can be found in “Data properties and evaluation metrics” section

PRICE [31] applies extension-based strategies for contig assembly. Using the seed reads as initial contigs, PRICE can be readily used to perform targeted viral assembly from metagenomic data. Therefore, the results of TAR-VIR was also benchmarked against PRICE’s results, as shown in Table 3. PRICE produced one long contig (similar N50 to ours) for the most abundant haplotype. Thus, its genome coverage is only about 20%.

**Table 3:** Assembly results on SARS-CoV metagenomic data for TAR-VIR and PRICE

	Tool	# Contigs	N50	Genomes Covered (%)	Mismatch (%)
Bowtie2 L	TAR-VIR	7	29,676	98.9	0.0
	PRICE	1	29,749	20.0	1.7
BWA B:1	TAR-VIR	6	29,706	99.5	0.0
	PRICE	1	29,750	20.0	1.66

### Exp2: characterizing hepatitis viruses from the human plasma data

In this experiment, TAR-VIR is tested on a real metagenomic data set, which was sequenced from the plasma of 19 antiretroviral-treated HIV patients (SRR2083204) [4]. The samples were pre-amplified by random RT-PCR amplification (RA) for both viral RNAs and DNAs and then sequenced by Illumina Miseq, producing about 23 million reads. All these samples contain low levels of HIV because of the antiretroviral treatment. But it may contain other human pathogens. In our study, we focused on identifying hepatitis viruses. Although our pipeline is designed to tackle the challenges of characterizing RNA viral quasispecies, we also include in the references DNA hepatitis viruses such as HBV.

#### Preprocessing

The raw data set contains reads that come from varied sources: human, bacteria, phages, etc. The reads of the target viruses comprise less than 30% of the entire data set. Since the primary focus is human viruses, removing those reads from the host (human), bacteria, and phages is ideal before pathogen detection. Following canonical quality control and trimming, we used bamtagger [32] to remove human reads, and Bowtie2 to remove reads from bacteria and phage by aligning reads against their reference genomes. The remaining reads were corrected by error correction tool Karect [33]. After the preprocessing step, 8,145,722 reads were left.

#### Recruited reads by TAR-VIR can improve the performance of de novo assembly

In the first step, we conducted read mapping to obtain the seed reads. Both BWA and Bowtie2 could be used. However, although BWA aligned more reads, many reads yielded only short local alignments and are unlikely to be sequenced from the target viruses. Using these reads as seeds tends to cause contamination during the read recruitment stage. For example, when BWA (“-B 8, -A 1") was used to generate the seed set, roughly 3.5 million reads were recruited, while a portion of them can be aligned to other genomes (such as phages). Although BWA’s output can be processed to remove local alignments, the seed set can be more reliably produced using Bowtie2’s output. Therefore, Bowtie2 was chosen as the aligner for all real data experiments.

We downloaded the reference genomes of HBV (NC_003977.2), multiple genotypes of HCV (NC_009827.1, NC_009823.1, NC_009825.1, NC_030791.1, NC_004102.1, NC_009826.1, NC_009824.1), and human pegivirus (HPgV, NC_001710.1) from the viral genome database of NCBI. The preprocessed reads were then aligned to the references under mismatch rates of 5%, 10%, 15%, and 20%, respectively. These initially aligned reads were used as the seed read sets. Although there are multiple genotypes for HCV, only genotype 1 has a decent amount of aligned reads. Other genotypes have less than 50 reads mapped. Thus, to produce a reliable evaluation of the assembly results, only the results of HCV genotype 1 were used. The numbers of reads before and after read recruiting are shown in Table 4.

**Table 4:** Overlap extension results using different seed set *R*_0_

Align mismatch	Seed #	Recruited #	Align mismatch	Seed #	Recruited #
5%	21,925	200,650	10%	67,973	222,065
15%	162,454	263,029	20%	294,448	340,705

‘#’ represents ‘number’. The shaded regions in this table and Table 5 highlight the case where less recruited reads can produce better assembly results than aligned reads only

As this is a real metagenomic data set without known ground truth of the viral haplotypes, the evaluation metrics for read recruiting are different from the simulation data set. We cannot evaluate whether every recruited read is correct because its originating location is unknown. Thus, instead of evaluating the depth and genome coverage for each haplotype, we focus on evaluating whether using recruited reads can improve the performance of genome assembly.

Therefore, both the aligned reads and the recruited reads from TAR-VIR were assembled by de novo assembly tools, and the results were compared in Table 5. The assembly results demonstrate that the reads recruited by TAR-VIR usually improve the assembly results by producing longer contigs and higher genome coverage for PEHaplo, SGA, and SPAdes. The improvement is not simply due to the increased number of reads after the recruitment stage. For example, according to Table 4, by using 15% mismatch rate, the recruited reads are less than the aligned reads under 20% mismatch rate (263,029 vs. 294,448). However, the assembly results using the recruited reads are better than or comparable to the results using the aligned reads for all the assembly tools. Among the four assemblers used, PEHaplo of TAR-VIR and SPAdes produced good results with large N50 and high genome coverage. SGA generated larger number of contigs with low N50 value. While we have tried the best parameters for SAVAGE based on our empirical experience, its results are not consistent with the other three tools. Better parameters may exist for SAVAGE to produce better results. However, the long-running time and high memory usage of this tool made continuing to tune the parameters difficult.

**Table 5:** Assembly evaluation results on aligned and recruited reads using the genomes of HBV, HCV, and HPgV as references

Align	Tool	Bowtie2 aligned			Bowtie2 Recruited		
		Contig #	N50	Genome cov. (%)	Contig #	N50	Genome cov. (%)
5%	TAR-VIR	11	920	27.3	97	3643	82.3
	SGA	14	645	26.8	63	675	68.4
	SPAdes	5	1177	27.6	15	3636	79.6
	SAVAGE	13	698	21.6	49	806	40.4
10%	TAR-VIR	61	794	67.4	31	2635	84.0
	SGA	26	663	56.4	72	706	69.5
	SPAdes	15	1251	65.4	19	3373	79.3
	SAVAGE	30	631	40.6	32	915	26.5
15%	TAR-VIR	97	939	80.9	14	3579	83.1
	SGA	56	617	57.7	74	722	70.2
	SPAdes	20	1689	77.6	16	3986	81.0
	SAVAGE	32	639	29.9	24	999	27.6
20%	TAR-VIR	38	1852	84.5	77	5678	86.4
	SGA	78	661	59.5	374	537	64.5
	SPAdes	23	2,710	83.8	10	4830	84.6
	SAVAGE	19	671	19.1	15	823	5.5

‘cov.’ is the abbreviation for ‘coverage’. The default assembly component in TAR-VIR is PEHaplo

#### Comparison with reference-based and extension-based assembly methods

With the reference genomes available, reference-based tools can be applied for viral metagenomic data analysis. Therefore, we also benchmarked TAR-VIR against reference-based haplotype reconstruction tools including Haploclique [19], drVM [16], and ViQuas [20]. VirusTap [17] can also conduct reads classification and then assembly. While we were planning to compare TAR-VIR with VirusTap, a large data set could not be uploaded to the website-based VirusTap. In addition, about 3000 jobs were waiting at the website. Therefore, the results from VirusTap could not be reported.

The reads aligned with the mismatch rate of 15% were used as input for Haploclique and ViQuas. For drVM, the reference genomes were built from human viruses, and it ran on the raw fastq files (with simple quality control and trimming) dumped from SRA file with default parameters. The seed read set of TAR-VIR were also the reads mapped with 15% mismatch rate. The assembly results are shown in Table 6.

**Table 6:** Assembly results comparison with reference- and extension-based methods

Tool	Contig #	N50	Genome cov. (%)	Tool	Contig #	N50	Genome cov. (%)
TAR-VIR	14	3579	83.1	ViQuas	396	9646	100.0
Haploclique	50,419	304	71.1	drVM	413	829	81.9

The results show that TAR-VIR performs better than Haploclique and drVM by producing fewer but longer contigs with higher genome coverage. With the complete and also the likely “true” virus genomes as the reference, ViQuas has produced near-complete genomes. However, it produces almost 400 contigs with similar lengths (full genomes), indicating a high probability of overestimation of the haplotypes. Since the ground truth of the actual number of haplotypes in this data set is unknown, we intended to test this hypothesis using a dataset with known haplotypes. Therefore, we tested ViQuas on the SARS-CoV simulated data set with 5 haplotypes. It reported 113 contigs, each covering 99.98% of the genome with high mismatch rate (> 9.0%). Thus, the long contigs produced by ViQuas are not likely the true haplotypes.

Similar to SARS-CoV data, we also benchmarked our results against the extension-based tool PRICE. The initial contigs of PRICE were also the reads mapped with 15% mismatch rate. PRICE generated 164 contigs, with a N50 value of 791, and genome coverage of 87.3%. PRICE’s results have a slightly larger genome coverage but a much smaller N50 value comparing to TAR-VIR.

#### Assembling the whole data set directly

As SGA and SPAdes are highly efficient and have been used by various virus analysis pipelines, it may be possible to directly apply them to all the preprocessed reads for recovering the three viruses ((HBV, HCV genotype 1, and HPgV). Thus, we applied SGA and SPAdes to the preprocessed reads. The assembled contigs were compared with the reference genomes of the three viruses. SGA took about 1 h to finish. It generated 2659 contigs, from which 123 contigs can be aligned to the three viruses with the similarity threshold of 90%. The 123 contigs can cover 42.36% of the reference genomes. SPAdes failed to report the results within 24 h. The results from SGA verified that although the preprocessed data set contains all the reads from the target viruses, the sheer data size and the low proportion of the three viruses make generic assembly difficult. Meanwhile, assembling a large data set consumes significant computing resources.

### Identifying viruses containing target genes

In some situations, the researchers are only interested in the viral genomes containing a partial or complete gene. In these cases, it is difficult for existing reference-based virus identification tools to construct the whole viral genome. Here, we demonstrate that with the overlap extension method, the most of a genome can be built from a partial gene reference.

In this experiment, we show that with a non-complete gene sequence of length 1073 bp for HPgV as reference, most of the genome can be assembled. The reference sequence (Sequence name: 10MYKJ037) was downloaded from Virus Pathogen Database and Analysis Resource (ViPR) [34], which is a partial coding DNA sequence (CDS) of HPgV isolated from Malaysia in 2010. The total length of HPgV genome is 9392 bp. From the results of our previous experiments, overlap extension from aligned reads under mismatch rate of 15% was able to recruit adequate reads while keeping away unreliable reads as seeds. Therefore, we aligned the raw reads to this CDS reference by allowing mismatch rate of 15%, from which 19,714 reads were aligned. ViQuas and drVM were used to assemble the aligned reads. However, ViQuas could only produce contigs similar to the short CDS sequence. In addition to provided CDS reference, drVM also downloaded references from Internet. It correctly recognized the HPgV but failed to produce any contig. The results confirm that reference-based methods do not apply in this case. With overlap cutoff of 150 bp, 118,339 reads were recruited from the overlap extension step. They were then assembled by PEHaplo of TAR-VIR, SGA, SPAdes, and SAVAGE, as shown in Table 7.

**Table 7:** Assembly results on recruited reads with a partial CDS sequence for HPgV as reference

Tool	Contig #	N50	HPgV cov. (%)	Tool	Contig #	N50	HPgV cov. (%)
TAR-VIR	5	7959	86.0	SPAdes	6	8957	94.0
SGA	41	591	49.0	SAVAGE	35	580	49.5

While the length of reference strain being only 11.42% of the whole HPgV genome, the contigs assembled from recruited reads by TAR-VIR and SPAdes are able to cover the nearly complete genome. The results reveal that even with a gene/CDS sequence as reference, sufficient reads can still be collected to construct the virus at the whole genome level. As there is only one target virus, SPAdes produced the best results. Applying SPAdes to the whole human plasma data set failed to finish on the cluster after 24 h, but by using recruited reads, SPAdes can produce better assemblies with the minimum amount of resources.

### Computational time and memory usage

We evaluated the time and memory usage of TAR-VIR on the real human plasma data. After preprocessing, 8,145,722 reads were left. The data size is 2.9 GB, and the total length of the sequences are 2,447,741,491 bp. To reduce memory usage, the raw data was split into 5 parts, with 5 BWTs being built for the whole data. The splitting process is embedded in our program, and the number of segments can be set by users. For each partition, the file sizes for the BWT, Occ array, and the read ID array are 490M, 200M, and 13M, respectively. The total size of built indexes is 3.5 GB. The detailed time and memory usage for the overlap extension is shown in Table 8 below. A user can load each partition separately to reduce the memory usage. In that case, the memory usage is about the size of each partition. In addition, we may further reduce the memory usage of the recruiting process by applying more compact implementation of the BWT [35].

**Table 8:** Time and memory usage for overlap extension and assembly on viral metagenomic data from human plasam

		Time (m: minutes, h: hours)				Memory (GB)			
Mismatch rate		5%	10%	15%	20%	5%	10%	15%	20%
Overlap extension	Building index	127m	127m	127m	127m	2.4	2.4	2.4	2.4
	Recruitment	8m	14m	20m	23m	3.5	3.5	3.5	3.5
De novo Assembly	TAR-VIR	5m	7m	18m	20m	2	2.8	3.5	4
	SGA	2m	2m	3m	5m	0.9	0.9	0.9	0.9
	SPAdes	5m	5m	6m	8m	1	1	1	1
	SAVAGE	2h	8h	14h	18h	31	45	51	59
Reference-based assembly	HaploClique			33h				5	
	Viquas			72h				4	
	drVM			23m				1	
Extension-based assembly	PRICE			1h46m				5	

The de novo assembly time and memory usage were evaluated on recruited reads based on mismatch rate from 5% to 20%. HaploClique, Viquas, drVM and PRICE were applied only on recruited reads based on mismatch rate of 15%

For all the assembly tools, Table 8 recorded their running times and memory usage on the recruited reads. Our experiments have shown that applying the assembly tools to the recruited reads can be more efficient and accurate. In addition, some tools cannot return results when being applied to the whole metagenomic data.

All the experiments were tested on an MSU HPCC CentOS 6.8 node with Two 2.4Ghz 14-core Intel Xeon E5-2680v4 CPUs and 128GB memory. We used 4 threads for the assembly component of TAR-VIR, 8 threads for SGA, 16 threads for SAVAGE, 8 threads for PRICE, 1 thread for Haploclique and ViQuas, and 2 threads for drVM.

## Discussion

Our results on both simulated and real metagenomic data have demonstrated the utilities of TAR-VIR in viral read classification. In this section, we discuss three practical issues related to using TAR-VIR.

TAR-VIR requires seed reads as input, which can be constructed using read mapping against homologous genomes or genes of interest to users. We compared several modes of popular read mapping tools including Bowtie2 and BWA. It is true that using local alignment mode for read mapping can produce a larger seed set. However, the size of the recruited reads depends more on the sequencing depth rather than the number of seed reads. Our experimental results also show that even a small seed set can recruit sufficient reads to cover the targeted genomes if the reads from these genomes share overlaps larger than the threshold. The quality of the seed set is more important to the performance of the read recruiting step. The local alignment mode may introduce contamination from unrelated species, leading to a read set containing non-targeted genomes. Thus, we recommend to use glocal mode to construct the seed set. If local mode is chosen, users should screen the read mapping results using alignment length and score.

A related issue is the choice of the overlap threshold. We have provided guidance on choosing appropriate overlap cutoffs based on the analysis of the common string sizes between different species. However, when the sequencing coverage is low, a smaller overlap threshold should be used. This is a similar problem to choosing overlap threshold or kmer size for assembly. Users can start with the recommended overlap threshold and test smaller ones gradually. If the size of the recruited set increases significantly at one step during the test, the process should stop.

TAR-VIR has a modular structure and the default assembly tool is PEHaplo. Users can replace PEHaplo with other assembly tools depending on the applications. For example, TAR-VIR can be extended to find bacterial species containing genes of interest to users. Thus, an assembly tool designed for bacterial species can replace PEHaplo.

## Conclusions

In this study, we presented a novel pipeline for viral reads classification and strain-level assembly from viral metagenomic data named TAR-VIR. When a virus in a metagenomic dataset is only remotely related to a characterized virus in public databases, our pipeline can be applied to first classify the reads belonging to these viruses and then conduct strain-level assembly. Or if a user is interested in detecting a virus that contains a given gene, our method can be employed to recover the whole genome of the gene-containing virus.

We also made contributions by conducting careful analysis of the common region sizes between and within viral quasispecies. These analyses laid the foundation for using overlap detection to classify reads of the same quasispecies without introducing contamination. Our unique implementation of the indexing structure also make our method economical in both memory and CPU usages.

We demonstrated the tool’s utilities on a simulated viral metagenomic data containing SARS-Cov and a real viral metagenomic data set sequenced from human plasma. The simulated data enables us to evaluate the performance of read classification to the resolution of each single read. It shows that TAR-VIR can successfully classify enough reads to cover the whole genome. In addition, it produced contigs covering five different haplotypes.

On the human plasma data, we were able to enrich enough reads from the target viruses for downstream assembly even with a small seed read set. With a partial CDS sequence for HPgV as reference, TAR-VIR was able to produce near complete genome assemblies. The results clearly showed the effectiveness of TAR-VIR. In summary, TAR-VIR provides complementary functions to existing virus detection tools when the quality or complete references are not available.

## Methods

Following a stand-alone error-correction step, our pipeline performs the following three steps. *First*, we construct the set of “seed reads” by mapping the reads against provided reference sequences, which could be sequenced genomes or functional sites such as genes. All the reads that can be mapped to the reference constitute the set of “seed reads”. The read mapping process can be conducted using existing tools such as Bowtie2 [25]. *Second*, we recruit reads that form significant overlaps with the seed reads. Newly recruited reads will be added to the seed set. This process will iterate until no new reads can be recruited. *Third*, we conduct strain-level assembly using the reads identified in the second step.

### Two scenarios

**Fig. 2:**
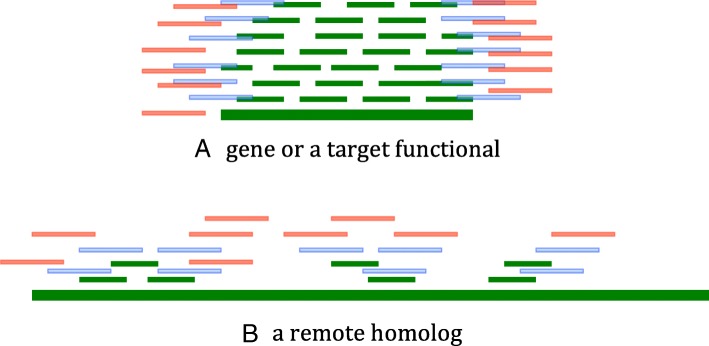
Two scenarios. **a**. The reference is a gene or a functional site (long green bar). The reads are represented by short lines. Short green lines can be mapped to the reference sequence and define the set of seed reads. The first iteration of overlap detection will identify new reads (blue lines) overlapping with the seed reads. The second iteration of overlap detection will identify more reads (red lines). **b**. The reference is a remotely related genome (long green bar). The seed reads can be mapped to the reference genome and are represented by short green lines. Two iterations of overlap detection will recruit new reads (blue lines and red lines, respectively)

The above pipeline is visualized for two scenarios in Fig. 2. In scenario 1, users are trying to detect viruses that contain a functional site, such as a gene. Unlike the well-studied gene-centric assembly in metagenomic data, our goal is to recover the whole genome that contains a particular gene. In this method, the gene is provided as a reference, and reads mapped to it are the seed reads. Overlap detection is then applied to recruit more reads that belong to the same viruses as the seed reads. The read recruitment process is presented in Fig. 2a.

In scenario 2, the goal is to identify viruses that lack quality reference genomes. This is particularly important for host-switching viruses, which may not always conserve high sequence similarity with their related peers in other hosts. For example, SARS-CoV shares about 80% of sequence identity with the bat coronavirus according to BLAST [36]. And the identity is lower than 50% at different loci. Thus, conventional read mapping methods cannot capture all reads from the targeted viruses when they lack high similarity with the available references. Figure 2b presents the process of identifying reads of the target virus with a remotely related virus as the reference. Although the mapped reads are scattered along the reference genome with low coverage, sufficient reads belonging to the target virus can be recruited through overlap detection.

### Validity of read recruitment using overlap detection

In this section, we will conduct careful analysis to examine whether using overlaps will be both sensitive and accurate for classifying reads in the same quasispecies. An ideal read recruiting process should only capture the reads from the targeted viral quasispecies. If many microbes share long common regions with the targeted viruses, the overlap extension will recruit a large number of reads from unrelated species. Therefore, we computed the sizes of longest common substrings (LCSs) between different viruses. The LCSs between viruses and other microbial species were also examined. The details for LCS calculation can be found in Additional file 1: Section 1. The results are shown in Additional file 1: Figure S1(A-C).

In summary, the sizes of LCSs between different viral genomes or between human viruses and bacteria are usually smaller than 100 bp. LCSs longer than 100 bp are mostly between viruses from the same genus or different genotypes of the same virus. For example, Vaccinia virus and Variola virus share an LCS of 469 bp, and HCV genotype 7 and HCV genotype 5 share an LCS of 154 bp.

Meanwhile, it is also necessary to evaluate whether reads belonging to the same quasispecies can be recruited using overlap detection. As the characterized haplotypes for different RNA viruses are very limited, instead of computing the LCS using available data, we estimated the LCSs within a quasispecies using a probability model. With the mutation rate *μ* at each base during virus replication, the probability distribution of LCS length between two viral strains that are *n* generations apart can be calculated with dynamic programming [37]. As an example, the probability distribution of LCS sizes between two HIV strains is shown in Additional file 1: Figure S1(D).

The result reveals that the LCSs between different haplotypes of the same viral population are usually much longer than LCSs between different viruses or an Illumina read size, which is about 250 bp for MiSeq reads in our experiments. Thus, even with the initial seed reads aligned to only one haplotype, the reads of other haplotypes can be recruited through the long common regions shared by different haplotypes. The reads sequenced from the common regions act like baits to recruit reads from different haplotypes.

In order to use the LCS size distribution to provide guidance for overlap cutoff choice, we plot the ROC curve using the data from Additional file 1: Figure S1. In the ROC curve, the true positive rate (TPR) for a given size *l* defines the probability that two strains within a quasispecies have a LCS at least *l*. The false positive rate (FPR) for a given size *l* defines the probability that two different microbial species have an LCS at least *l*. TPR can be derived using the area size in Additional file 1: Figure S1(D) while FRP can be computed using Additional file 1: Figure S1(A)-(C). The final ROC curve has a AUC close to 1, which is expected because of the small overlap between the LCS values for haplotypes and different species. The ROC curve is shown in Additional file 1: Figure S2. Meanwhile, because there are 142,021,586 pairs of viruses vs. other microbes, which can lead to a very small FPR value, we thus also show the actual number of virus-vs-other pairs with LCS size above *l* in Additional file 1: Figure S3. For example, there are 86 virus-vs-other pairs (out of 142,021,586) with LCS value above 100 bp. In practice, not all these microbes live in the same niche and thus are included in the same metagenomic data set. Thus, this is the worst-case contamination analysis using characterized genomes. Additional file 1: Figures S1-S3 show that choosing overlap size above 100 can lead to high sensitivity and near zero FPR. With a bigger cutoff, the FPR can be further reduced. But if the cutoff is too big, reads from low coverage regions cannot be recruited.

#### Chimeric reads

**Fig. 3:**
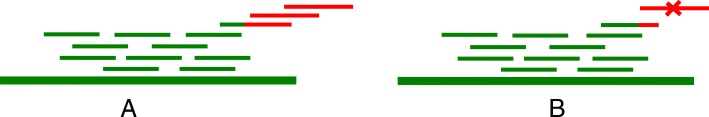
Chimeric reads may introduce contamination. The reference is a gene or a functional site (long green bar). The reads are represented by short lines. Green reads are sequenced from the reference. Red color represents sequences from another species. **a**. When the overlap cutoff is small, a chimeric read can be extended and thus recruits reads from other species. **b**. When the overlap cutoff is bigger than half of the read size, a chimeric read could be recruited but will not be extended in the following iterations

One recent study revealed that chimeric reads, which contain sequences from more than one species, can be generated in vitro during the preparation of high-throughput sequencing libraries [38]. These chimeras may have overlaps with more than one species, thus introducing contamination from the host or unrelated microbes. Figure 3a illustrates how contamination can be introduced via chimeric reads. In our experiments, we set the overlap threshold longer than half of the read size to prevent recruiting these chimeric reads or extending from them. To justify our choice of the overlap threshold for preventing contamination via chimeric reads, consider a chimeric read that is a concatenation of the sequences from the target virus and another species. There are two cases. In the first case, if the region from the target virus is shorter than half of the read size, this chimeric read will not be recruited. In the second case, if the region from the target virus is longer than half of the read size, this chimeric read will be recruited. However, as the other part of this chimeric read must be shorter than half of the read size (i.e. overlap threshold), this read will not be extended in the next iteration. Figure 3b shows this case. If the chimeric read contains regions from more than two species, it becomes harder for this read to form an overlap above the cutoff with reads from the target virus.

In our experiments, we set the overlap cutoff as 150 for all reads of 250 bp long.

#### Sequencing errors

Sequencing errors will shorten overlaps between reads and may prevent recruiting all reads belonging to the same quasispecies. To recruit sufficient reads for assembly, we can construct either approximate overlaps by allowing mismatches/gaps or exact overlaps on error-corrected reads. Considering the risk of contamination by approximate overlap detection, we chose to use stand-alone error correction tools paired with exact overlap detection. The default error correction tool in our pipeline is Karect [33]. Low sequencing depth will lead to small overlaps and thus affects the performance of read recruitment. There is a possibility that the reads from regions of low coverage cannot be recruited and assembled.

### Read recruiting

In order to describe the algorithm, we formally define overlap. Let *r*_*i*_ and *r*_*j*_ be two reads. If there is a proper suffix of *r*_*i*_ that is the prefix of *r*_*j*_ or vice versa, *r*_*i*_ and *r*_*j*_ form an overlap. In practice, we will also account for the overlaps formed by *r*_*i*_ and *rj*′s reverse complement. There are a few data structures and methods available for efficient overlap detection [28, 39]. We apply the methods with BWT and FM-index [28] for efficient search. In the first step, all reads are concatenated into a single sequence *T*[1..*n*] using *$* as a delimiter, where *n* is the number of reads in *T*. Then, multi-key “quicksort” is applied to sort all the suffixes of *T* for constructing a generalized suffix array *SA*(*T*) [40]. Then *BWT*(*T*) can be constructed using the following equation, where *BWT*[*i*] and *SA*[*i*] are abbreviated representations of *BWT*(*T*)[*i*] and *SA*(*T*)[*i*], respectively.

1 $$\begin{array}{@{}rcl@{}}  BWT[i]=\left\{ \begin{array}{lcl} T[SA[i]-1], & \text{if}\quad SA[i]>0\\ \$, & \text{if}\quad SA[i]=0\\ \end{array} \right. \end{array} $$

With *T* and *BWT*(*T*), the backward search can be used to detect overlaps between a query read and all other reads. After matching *τ* (the overlap threshold) characters, we search for the delimiter ‘ *$*’ to find the prefixes overlapping with the query’s suffix.

#### Unique implementation strategies

Although there are available implementations of the BWT-based overlap detection, ours differs from the existing ones in the following aspects. The first difference is the storage of the read ID information. For a constructed BWT and a query, the output of the backward search is the set of reads (i.e., their IDs) that form overlaps with the query. Theoretically, different reads can be distinguished by appending unique delimiters at the end of each read. In our implementation, we use ‘ *$*’ as the delimiter for all reads. The read ID array *RID* is created only for suffixes starting with ‘ *$*’. This works because the backward search algorithm needs to retrieve the read ID in the final step, where the character to search is ‘ *$*’. This modification reduced the size of *RID* from |*T*| integers to *n* (number of reads) integers, where *T* is roughly the product of *n* and the read size.

### Iterative search

Overlap detection will be iteratively applied to recruit reads sequenced from targeted viruses. Let *R*_0_ be the set of seed reads that can be mapped to given reference sequences (i.e., seed read set). First, *BWT*(*T*) for *T* is built. The seed reads in *R*_0_ are used as queries to *BWT*(*T*). Then newly identified reads that overlap with the seed reads will be used as new queries to the BWT. The iterations will continue until no new reads can be retrieved. Its pseudocode is described in Additional file 1: Section 2.

#### Running time and memory usage

In the above pipeline, once the BWT is constructed, the suffix array will be deleted. The running time of suffix array and BWT construction is linear to |*T*|. The memory usage of BWT is the product of |*T*| and the size of each character and thus is linear to |*T*|. The memory usage of the *RID* is the product of *n* and the size of saving a read ID.

When creating BWT for all reads becomes too expensive, our program supports distributed construction of the BWT and FM-index for large input. Specifically, the program can automatically partition input data into multiple smaller files. BWT is then constructed for each divided data set. The read overlap detection can be run in parallel for each BWT. The identified reads are combined and used as the query for the next iteration of read recruitment. In this case, the largest memory footprint is determined by the size of each divided read set. By default, the number of partitions is five. This number can be modified by users.

### Strain-level assembly

The final outputs of our program are assemblies of viral strains. All recruited reads will be used as input to assembly programs. As our program has a modular structure, this step can be executed by any assembly tool chosen by the users. By default, we include in the package our in-house developed tool PEHaplo [37] for viral haplotype reconstruction. PEHaplo does not require any reference sequences and conducts strain-level assembly using paired-end reads. For the input paired-end reads, PEHaplo constructs a paired-end overlap graph, which augmented standard overlap graphs by adding edges connecting nodes that can form ends of read pairs. Then, a greedy path finding algorithm is applied to search for the paths with the best supports from paired-end reads, where the supports are quantified by the number of contained read pairs and also their distances. For the detailed algorithm and implementation of PEHaplo, we refer the readers to the manuscript.

## Additional file

Additional file 1 Supplementary information for LCS sizes, iterative search, complete assembly results for SARS-CoV data, and tool commands. (PDF 790 kb)
