# Supplementary material for: TAR-VIR: a pipeline for TARgeted VIRal strain reconstruction from metagenomic data
Source: BMC Bioinformatics. 2019 Jun 4;20:305. doi: 10.1186/s12859-019-2878-2 (PMC6549370; doi:10.1186/s12859-019-2878-2)
Supplement: Supplementary file 1 — Supplementary information for LCS sizes, iterative search, complete assembly results for SARS-CoV data, and tool commands. (PDF 790 kb) [file 12859_2019_2878_MOESM1_ESM.pdf]

BMC Bioinformatics

Supplementary

TAR-VIR: a pipeline for TARgeted VIRal strain reconstruction from metagenomic data

Jiao Chen [1], Jiating Huang [2], and Yanni Sun [3,*]

[3]Electronic Engineering, City University of Hong Kong, Hong Kong, China SAR.

1 Sizes of common regions between human viruses and other microbial species

To evaluate the similarity between different microbes, we calculated the sizes of LCSs between human viruses and other microbial genomes. As bacteria infect humans as well as viruses, the sizes of LCSs between human viruses, human vs. non-human viruses, and human viruses vs. bacteria were calculated. The virus reference genomes were downloaded from NCBI Viruses (https://www.ncbi.nlm.nih.gov/genome/viruses/). To date (June 2018), there are in total 7,456 complete viral genomes, of which 481 have human as the natural host (denote as human viruses). The human bacterial reference genomes were downloaded from Human Microbiome Project (HMP) on NCBI. In total, 2,314 bacterial reference genomes were downloaded.

As there is a large number of microbial species available, we conducted LCS search for available microbial genomes by constructing generalized suffix array and the corresponding longest common prefix (LCP) array (Gusfield, 1997). *First*, we build a generalized SA (Rajasekaran and Nicolae, 2014). *Then*, the LCP array, which contains LCPs between each two adjacent suffixes, can be calculated in linear time (Kasai *et al.*, 2001; Kärkkäinen and Sanders, 2003). By definition, the LCS for each two sequences is the maximum LCP between all pairs of suffixes from the two sequences. The following lemma is employed in order to avoid checking all the LCP values between two sequences. For the suffix starting at $SA[i]$, the LCP between $SA[i]$ and $SA[j]$ $(j > i)$ is no less than the LCP between $SA[i]$ and $SA[k]$ if $k > j$. With this property and a user-defined LCS cutoff, the LCP calculation between $SA[i]$ and all other suffixes after i can be calculated in constant time. The overall time complexity is $O(N)$.

The results of the LCS histograms are shown in Figure S1(A-C). One may also examine whether the read recruitment process can incur contamination by using simulated or real sequencing data. However, the empirical studies using real data are limited to the viruses in the samples. Meanwhile, producing simulated sequencing data for all microbes is not practical. Using suffix-array based LCS computation allows us to obtain a more comprehensive view of the common regions between different microbes.

We also compared the sizes of the LCSs between different microbes with the ones within a viral population. As the characterized haplotypes for different RNA viruses are very limited, instead of computing the LCS using available data, we estimated the LCSs within a quasispecies using a probability model and dynamic programming (Chen *et al.*, 2018). With the mutation rate of 3e-5 at each base during virus replication, the probability distribution of LCS length between two HIV strains that are n generations apart were calculated and the distribution of LCS probabilities is shown in Figure S1(D).

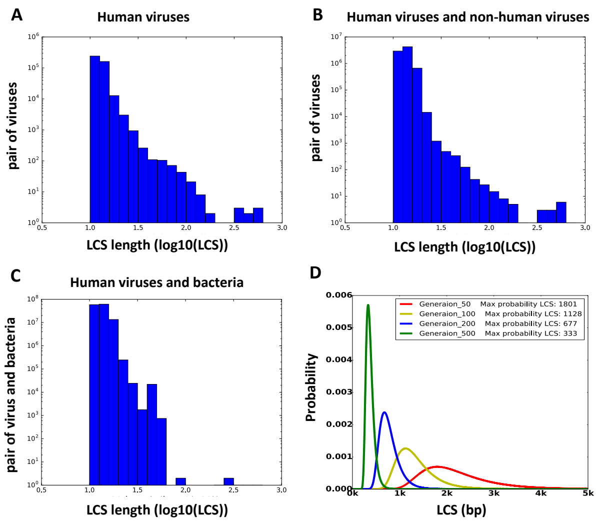

Fig. S1. Histogram of the LCS sizes between human viruses (A), between human viruses and non-human viruses (B), and between human viruses and bacteria (C). The x-axis is the $log10$ of the LCS length. The y-axis is the number of pairs within the given range of LCS size. Only LCSs that are longer than 10 bp are presented. (D) Probability distribution for LCSs between two simulated HIV strains that are 50, 100, 200, and 500 generations apart. The x-axis is the length of LCS, with a range from 0 to 10,000 bp. The y-axis is the corresponding probabilities for those LCS sizes.

To gain guidance on appropriate overlap cutoffs for extension, the ROC curve for LCS thresholds is plotted in Figure S2. As there are 142,021,586 pairs of virus-vs-other sequences, near zero FPR (false positive rate) values are generated for many LCS cutoffs. Thus we also presented the actual number of virus-vs-other sequence pairs with LCS above a given cutoff in Figure S3. For each LCS cutoff, the corresponding TPR (true positive rate) and the number of false positive pairs are illustrated using two axes. To compute the TPR and FPR, we define a positive case as two sequences from the same quasispecies. The negative case refers to a pair of genomes from two different species. Thus, given an LCS cutoff, the FPR can be computed as the percentage of negative cases with LCS above the cutoff in all 142,021,586 examined pairs. The TRP measures

© The Author . Published by Oxford University Press. All rights reserved. For permissions, please e-mail: journals.permissions@oup.com

the percentage of positive cases with LCS above the cutoff, which can be computed by sampling the distribution in Figure S1(D). In Figure S1(D), we have several distributions with different number of generations of replication. We generated 10^6 positive samples using the distribution with 100 generations. If one replication takes about 24 hours, this curve mimics the infection of about 3 months.

The AUC for the ROC curve is 0.999, which means sequences from different viral haplotypes in a quasispecies have different LCSs (larger) from sequences of different species. From Figure S3, when the overlap cutoff is between 100 bp to 500 bp, the FPR is close to 0 and TPR is close to 1. Users can choose overlap cutoff within this range but less than the read size. Taking into account the contamination from chimeric reads and the read size, an overlap threshold between 130 to 249 is advised. The actual cutoff still needs to be tested because different data sets have different coverage. For our experiments, the default cutoff is 150 for all MiSeq reads of 250 bp.

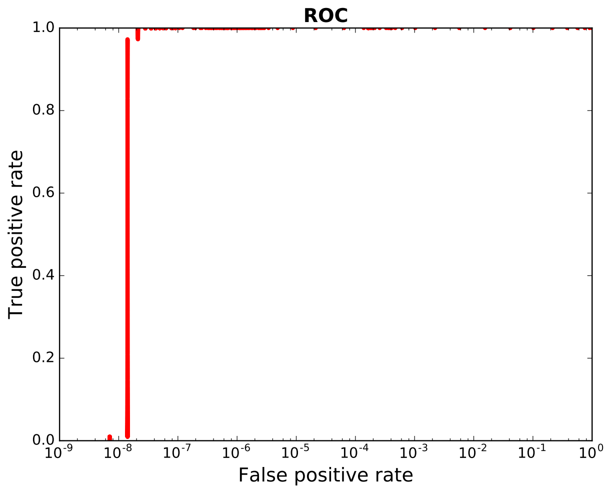

Fig. S2. The ROC curve for LCS cutoff. The x-axis is the false positive rate (FPR). The y-axis is the true positive rate (TPR).

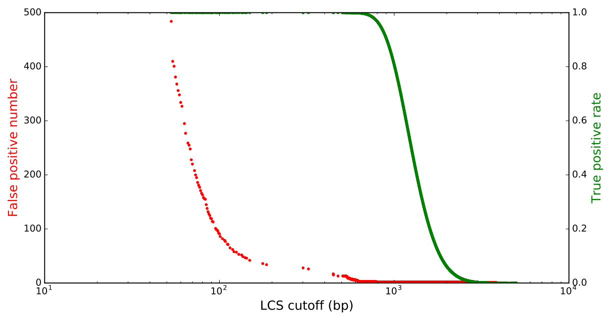

Fig. S3. False positive numbers and ture positive rates (TPR) for given LCS cutoffs. The x-axis is the value of LCS cutoff. The double y axes represent the value of FP and TPR, respectively.

2 Pseudocode for iterative search

Algorithm 1 Default mode: create BWT for all reads

Input: seed read set R_0, the input text T, the overlap threshold τ
Output: Reads that are sequenced from the targeted viruses

```
output ← R_0
R ← R_0
Create BWT and RID for T: BWT(T), RID(T)
while R not empty do
    Backward search on BWT(T) to find all reads that overlap with reads in R
    Save them to set R'
    output ← output ∪R'
    R ← R'
return output
```

3 Read recruitment and assembly results for simulated SARS-Cov data

The assembly results on SARS-Cov aligned and recruited reads are shown in Table S1.

4 Commands for running tools on SARS simulated data set

Input data: sars_meta_1.fa, sars_meta_2.fa or sars_meta_1.fq, sars_meta_2.fq or sars_meta_whole.fa
Reference: Bat_coronavirus.fasta

Overlap extension

1. Alignment
(1) Bowtie2

```
bowtie2-build -f Bat_coronavirus.fasta index/
     Bat_coronavirus

bowtie2 -x Bat_coronavirus -f -a --score-min L,0,-0.6 -
     t -p 16 -S bat_corona_align.sam" sars_meta_whole.
     fa

These are our recommended parameters for read mapping
     step.
```

(2) BWA

```
bwa mem -B 3 -A 1 -t 4 sars_meta_whole.fa >
     bwa_bat_corona_align.sam
```

2. Recruite reads with TAR-VIR's overlap extension component

```
build -f sars_meta_whole.fa -o sars_meta
overlap -S bat_corona_align.sam -x sars_meta -f
     sars_meta_whole.fa -c 150 -o sars_recruited.fa
```

Assembly

1. TAR-VIR's assembly compont

```
python pehaplo.py -f sars_meta_whole.fa -l 160 -l1 190
     -r 250 -F 600 -std 150 -correct yes -n 2 -t 4
```

2. SGA

Table S1. Assembly results on SARS-CoV aligned and recruited metagenomic data. N50 is defined as the maximal length so that all contigs above this length contain at least 50% of all the contig bases. Genome coverage is the percentage of the five haplotypes' genomes being aligned by at least one contig. Mismatch rate is the percentage of mismatches between the aligned contigs and the references.

Bowtie2 Aligned	Tool	# Contigs	N50	Genomes covered (%)	Mismatch rate (%)	Bowtie2 Recruited	Tool	# Contigs	N50	Genomes Covered (%)	Mismatch rate (%)
L,0,-0.3	PEHaplo	-	-	-	-	L,0,-0.3	PEHaplo	8	29,613	86.0	0.0
	SGA	-	-	-	-		SGA	14	26,301	86.0	0.0
	SPAdes	-	-	-	-		SPAdes	12	29,256	76.5	0.47
	SAVAGE	-	-	-	-		SAVAGE	19	21,389	85.9	0.0
L,0,-0.6	PEHaplo	12	374	2.96	0.0	L,0,-0.6	PEHaplo	9	29,643	89.6	0.0
	SGA	11	374	2.7	0.0		SGA	13	26,301	89.6	0.0
	SPAdes	5	384	1.3	0.10		SPAdes	17	16,445	80.3	0.29
	SAVAGE	12	362	2.9	0.0		SAVAGE	29	22,920	86.8	0.0
L,0,-0.9	PEHaplo	58	505	19.7	0.02	**L,0,-0.9**	PEHaplo	7	29,676	98.9	0.0
	SGA	56	505	20.1	0.03		SGA	13	26,729	98.9	0.0
	SPAdes	34	569	12.9	0.16		SPAdes	14	15,882	92.1	0.51
	SAVAGE	54	455	17.5	0.0		SAVAGE	22	12,445	97.0	0.0
L,0,-1.2	PEHaplo	91	568	32.8	0.0	L,0,-1.2	PEHaplo	7	29,698	99.5	0.0
	SGA	80	700	32.6	0.0		SGA	12	27,540	99.5	0.0
	SPAdes	52	695	23.5	0.13		SPAdes	17	12,822	92.6	0.4
	SAVAGE	74	500	25.2	0.0		SAVAGE	22	12,182	96.7	0.0
**BWA Aligned**	**Tool**	**# Contigs**	**N50**	**Genomes covered (%)**	**Mismatch rate (%)**	**BWA Recruited**	**Tool**	**# Contigs**	**N50**	**Genomes Covered (%)**	**Mismatch rate (%)**
B:8	PEHaplo	32	3,123	54.3	0.0	B:8	PEHaplo	6	29,687	99.5	0.0
	SGA	53	723	26.3	0.0		SGA	10	27,556	99.5	0.0
	SPAdes	34	2675	45.7	0.0		SPAdes	16	12,838	92.6	0.45
	SAVAGE	29	1709	23.4	0.0		SAVAGE	46	6,803	91.5	0.0
B:4	PEHaplo	38	5151	70.4	0.0	B:4	PEHaplo	7	29,680	99.8	0.0
	SGA	64	1177	41.6	0.0		SGA	9	27,563	99.8	0.0
	SPAdes	37	3170	64.4	0.12		SPAdes	15	13,494	94.6	0.49
	SAVAGE	43	1416	31.3	0.0		SAVAGE	40	9,300	91.3	0.02
B:2	PEHaplo	83	1,192	54.3	0.0	B:2	PEHaplo	6	29,683	99.7	0.0
	SGA	85	983	54.5	0.0		SGA	11	20,792	99.8	0.0
	SPAdes	66	1,012	43.8	0.11		SPAdes	19	17,766	87.9	0.46
	SAVAGE	53	763	28.1	0.0		SAVAGE	70	3,093	85.5	0.003
**B:1**	PEHaplo	84	1,192	55.1	0.0	B:1	PEHaplo	6	29,706	99.5	0.0
	SGA	85	1,027	56.5	0.0		SGA	18	12,638	99.5	0.0
	SPAdes	67	1,012	44.6	0.12		SPAdes	21	10,353	89.2	0.39
	SAVAGE	68	669	32.3	0.0		SAVAGE	56	5,140	89.3	0.0

```
#! /bin/bash -x

IN=sars_recruited.fa

# Parameters
SGA_BIN=sga

# Overlap parameter used for the final assembly. This
     is the only argument
# to the script
OL=150
ER=0.02

# The number of threads to use
CPU=8

# Correction k-mer value
CK=51

# The minimum k-mer coverage for the filter step. Each
     27-mer in the reads must be seen at least this
     many times
COV_FILTER=2
FK=51

# Overlap parameter used for FM-merge. This value must
     be no greater than the minimum
# overlap value you wish to try for the assembly step.
MOL=55

# Parameter for the small repeat resolution algorithm
R=10

# The number of pairs required to link two contigs into
      a scaffold
MIN_PAIRS=5
```

```
# The minimum length of contigs to include in a
     scaffold
MIN_LENGTH=350

# Turn off collapsing bubbles around indels
MAX_GAP_DIFF=0

# First, preprocess the data to remove ambiguous
     basecalls
$SGA_BIN preprocess -o virus.fa $IN

#
# Error correction
#
# Build the index that will be used for error
     correction
# As the error corrector does not require the reverse
     BWT, suppress
# construction of the reversed index
$SGA_BIN index -a ropebwt -t $CPU --no-reverse virus.fa

# Perform error correction with a 41-mer.
# The k-mer cutoff parameter is learned automatically
$SGA_BIN correct -k $CK --discard --learn -t $CPU -o
     reads.ec.k$CK.fa virus.fa

#
# Contig assembly
#

# Index the corrected data.
$SGA_BIN index -a ropebwt -t $CPU reads.ec.k$CK.fa

# Remove exact-match duplicates and reads with low-
     frequency k-mers
$SGA_BIN filter -x $COV_FILTER -k $FK -t $CPU --
     homopolymer-check --low-complexity-check reads.ec.
     k$CK.fa

# Merge simple, unbranched chains of vertices
$SGA_BIN fm-merge -m $MOL -t $CPU -o merged.k$CK.fa
     reads.ec.k$CK.filter.pass.fa

# Build an index of the merged sequences
$SGA_BIN index -t $CPU merged.k$CK.fa

# Remove any substrings that were generated from the
     merge process
$SGA_BIN rmdup -t $CPU merged.k$CK.fa

# Compute the structure of the string graph
$SGA_BIN overlap -m $OL -e $ER -t $CPU merged.k$CK.
     rmdup.fa

# Perform the contig assembly without bubble popping
$SGA_BIN assemble -m $OL -g $MAX_GAP_DIFF -r $R -o
     assemble.m$OL merged.k$CK.rmdup.asqg.gz
```

3. SPAdes

```
metaspades.py --meta --only-assembler -k 105,115,125 -s
      sars_recruited.fa -o sars_meta_output/
```

4. SAVAGE

```
pear -f sars_meta_1.fq -r sars_meta_2.fq -o
     sars_meta_join
```

```
savage --split 1 --min_overlap_len 120 -s singles.fastq
      -p1 paired1.fastq -p2 paired2.fastq -t 16
```

5. PRICE

```
init_contig.fa: sars_meta_whole.fa reads aligned on
     Bat_coronavirus.fasta
PriceTI -nc 5 -a 8 -fp sars_meta_1.fa sars_meta_2.fa
     600 -icf init_contig.fa 3 3 1 -o price_results.fa
```

References

Chen, J., Zhao, Y., and Sun, Y. (2018). De novo haplotype reconstruction in viral quasispecies using paired-end read guided path finding. *Bioinformatics*.

Gusfield, D. (1997). *Algorithms on strings, trees and sequences: computer science and computational biology*. Cambridge university press.

Kärkkäinen, J. and Sanders, P. (2003). Simple linear work suffix array construction. In *International Colloquium on Automata, Languages, and Programming*, pages 943–955. Springer.

Kasai, T., Lee, G., Arimura, H., Arikawa, S., and Park, K. (2001). Linear-time longest-common-prefix computation in suffix arrays and its applications. In *Annual Symposium on Combinatorial Pattern Matching*, pages 181–192. Springer.

Rajasekaran, S. and Nicolae, M. (2014). An elegant algorithm for the construction of suffix arrays. *Journal of Discrete Algorithms*, **27**, 21–28.
